# Scaling Up Stomatal Conductance from Leaf to Canopy Using a Dual-Leaf Model for Estimating Crop Evapotranspiration

**DOI:** 10.1371/journal.pone.0095584

**Published:** 2014-04-21

**Authors:** Risheng Ding, Shaozhong Kang, Taisheng Du, Xinmei Hao, Yanqun Zhang

**Affiliations:** 1 Center for Agricultural Water Research in China, China Agricultural University, Beijing, China; 2 National Center of Efficient Irrigation Engineering and Technology Research-Beijing, China Institute of Water Resources and Hydropower Research, Beijing, China; DOE Pacific Northwest National Laboratory, United States of America

## Abstract

The dual-source Shuttleworth-Wallace model has been widely used to estimate and partition crop evapotranspiration (*λET*). Canopy stomatal conductance (*G_sc_*), an essential parameter of the model, is often calculated by scaling up leaf stomatal conductance, considering the canopy as one single leaf in a so-called “big-leaf” model. However, *G_sc_* can be overestimated or underestimated depending on leaf area index level in the big-leaf model, due to a non-linear stomatal response to light. A dual-leaf model, scaling up *G_sc_* from leaf to canopy, was developed in this study. The non-linear stomata-light relationship was incorporated by dividing the canopy into sunlit and shaded fractions and calculating each fraction separately according to absorbed irradiances. The model includes: (1) the absorbed irradiance, determined by separately integrating the sunlit and shaded leaves with consideration of both beam and diffuse radiation; (2) leaf area for the sunlit and shaded fractions; and (3) a leaf conductance model that accounts for the response of stomata to PAR, vapor pressure deficit and available soil water. In contrast to the significant errors of *G_sc_* in the big-leaf model, the predicted *G_sc_* using the dual-leaf model had a high degree of data-model agreement; the slope of the linear regression between daytime predictions and measurements was 1.01 (R^2^ = 0.98), with RMSE of 0.6120 mm s^−1^ for four clear-sky days in different growth stages. The estimates of half-hourly *λET* using the dual-source dual-leaf model (DSDL) agreed well with measurements and the error was within 5% during two growing seasons of maize with differing hydrometeorological and management strategies. Moreover, the estimates of soil evaporation using the DSDL model closely matched actual measurements. Our results indicate that the DSDL model can produce more accurate estimation of *G_sc_* and *λET*, compared to the big-leaf model, and thus is an effective alternative approach for estimating and partitioning *λET*.

## Introduction

Accurate estimation of evapotranspiration (*λET*) is important in understanding terrestrial hydrological cycles because *λET* is the largest component in the terrestrial water balance after precipitation [1]. In agricultural production, improved estimation of crop *λET* is also needed to develop precise irrigation scheduling and enhance water use efficiency, as soil water depletion is mostly determined by the rate of *λET* [2], [3], [4]. However, direct measurement of *λET* is often difficult, costly and not available in many regions [5], [6]. Therefore, mathematical models are needed to estimate *λET* using readily measurable meteorological and environmental variables.

Vegetation transpiration (*T_r_*) and soil evaporation (*E_s_*), which are controlled by different biotic and physical processes, are the two major components of *λET*. Transpiration is strongly linked to crop productivity since it occurs concurrently with photosynthetic gas exchange [7]. Quantifying *T_r_* is also critical to accurately predict the response of crop functioning and physiology to changing climate [8]. Because the two separate processes occur simultaneously, there is no simple way to distinguish between them [9], [10].

Several models have been developed to calculate *λET* and separately estimate soil evaporation and transpiration [11], [12], [13]. Shuttleworth and Wallace [14] described a dual-source model with a resistance-energy combination, which could separately predict *T_r_* and *E_s_*, and is also sufficiently simple [8]. This model has been widely used and can also be used to gain an understanding of the interaction of biophysical and hydrological processes in the crop canopy [14], [15]. Determination of different resistances or conductances (the reciprocal of resistance) is necessary for its practical application. Specifically, canopy stomatal conductance (*G_sc_*) is often calculated by scaling up leaf stomatal conductance of the leaves acting in parallel while treating the canopy as one big-leaf, hereafter the big-leaf approach [8], [16].

The weakness of using the big-leaf approach is that the use of mean absorbed radiation can significantly overestimate *G_sc_*, especially in dense canopies, because the light response of stomata is non-linear [17], [18]. Moreover, the overestimation of *G_sc_* by the big-leaf approach often occurred when using total leaf area index (*LAI*) to scale up stomatal conductance. To mitigate the overestimation of *G_sc_*, researchers have used the effective or sunlit *LAI* (*LAI_e_*) instead of total *LAI* [16], [18]. But, the relationship between *LAI_e_* and *LAI* is empirical and varies with the vegetation type and solar radiation angle. Therefore, models of *G_sc_* should consider the non-linear response of stomata to irradiance as well as heterogeneous radiation profiles in the canopy. Beam and diffuse radiation penetrating the canopy must be considered separately due to differential attenuation in canopies, as should visible and near-infrared wavebands due to differential absorption by leaves [12], [19]. A multilayers model could account for the mechanism of radiation penetration [20], [21], but it is useless in practical applications [22]. Some studies indicate that radiation penetration within the canopy could be simplified by splitting the canopy into two fractions of leaves: sunlit and shaded [18], [23], [24]. Leuning et al. [25] used a multilayer model to show that photosynthesis from a canopy is closely approximated when calculated as the separate sums of sunlit and shaded fractions, weighted by their respective leaf area within the canopy. However, few studies have calculated *G_sc_* using the approach of sunlit and shaded leaves (hereafter, the dual-leaf approach) in the dual-source S-W model for estimating *λET*.

Soil surface resistance (*r_ss_*) is another key parameter for partitioning *λET* in the dual-source model because above-canopy *λET* is contaminated by flux from the soil substrate as variations in leaf area affect soil exposure, soil evaporation and absorbed radiation [14], [26]. To reduce soil evaporation in the field, the ground is often mulched with plastic film, and this technique is widely used in northwest China [10], [27]. But the effect of ground mulching on soil evaporation has not been taken into account when parameterizing *r_ss_* in the dual-source model. In this study, a dual-leaf model of *G_sc_* was developed by scaling up leaf stomatal conductance using the corresponding absorbed irradiance for the sunlit and shaded leaves separately, which overcomes the limitations of the big-leaf model. The dual-leaf model developed here was then incorporated into the dual-source model to estimate and partition *λET*. We evaluated the dual-source dual-leaf model (DSDL) by comparing the model estimations of *λET* with measurements taken in an irrigated maize field mulched with plastic film.

## Model Development for Evapotranspiration and Canopy Stomatal Conductance

### 1 Dual-source Evapotranspiration Model

The *λET* in the dual-source model was partitioned into two components, canopy transpiration (*λT_c_*) and soil evaporation (*λE_s_*) with a resistance network [14].
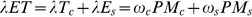
(1)

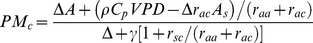
(2)

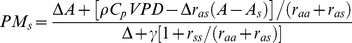
(3)

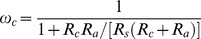
(4)

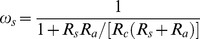
(5)

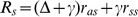
(6)

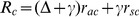
(7)

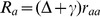
(8)where *PM_c_* and *PM_s_* are the terms similar to those in Penman–Monteith model for canopy transpiration and soil evaporation, respectively, and *ω_c_* and *ω_s_* are the weighting factors for the crop canopy and soil components, respectively. *λ* is the heat of water vaporization, *ρ* is air density, *C_p_* is the specific heat of dry air at constant pressure, *Δ* is the slope of the saturation vapor pressure curve, *γ* is the psychrometric constant, *VPD* is vapor pressure deficit, and *A* and *A_s_* are the total available energy and available energy for soil, respectively. *r_sc_* is canopy stomatal resistance, *r_ss_* is soil surface resistance, *r_ac_* is canopy boundary layer resistance, *r_as_* is soil boundary layer resistance between soil and vegetative canopy, and *r_aa_* is aerodynamic resistance between canopy source and reference height, respectively. The calculation procedures of the other resistances except *r_sc_* and *r_ss_* are given in Appendix S1.



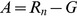
(9)

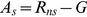
(10)

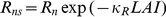
(11)where *R_n_* and *R_ns_* are net radiation above the canopy and at the soil surface, respectively, and *G* is the soil heat flux. The canopy extinction coefficient of net radiation, *κ_R_*, is dependent on leaf orientation and solar zenith angle (*ζ*) [17].
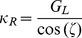
(12)where G_L_ is 0.5 for a spherical leaf angle distribution. ζ, the angle subtended by the sun at the center of the earth, is perpendicular to the surface of the earth and calculated as in Appendix S1.

The two components, *λT_c_* and *λE_s_* were now calculated along with the *VPD* at the canopy source height (*D_o_*).
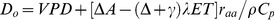
(13)

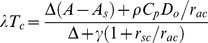
(14)

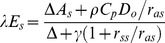
(15)


The measured *r_sc_* was obtained by inverting Eq. (14), with *λT_c_* calculated by the known or measured *λET* and *λE_s_*.
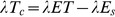
(16)

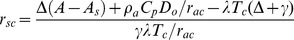
(17)


### 2 Irradiance within Crop Canopy

Incident PAR light above the canopy (*Q_o_*) was divided into diffuse (*Q_od_*) and beam irradiance (*Q_ob_*) through the fraction of diffuse radiation (*f_d_*).
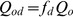
(18)

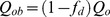
(19)


The *f_d_* was calculated from a simple model of atmospheric attenuation of radiation [17], [28].
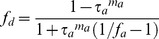
(20)where *τ_a_* is the atmospheric transmittance, *f_a_* is the forward scattering coefficient of PAR in atmosphere, and *m_a_* is the optical air mass, which can be calculated as follows.
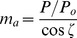
(21)where P is local atmospheric pressure and P_0_ is atmospheric pressure at sea level.

At a depth *ξ* in the canopy, three types of irradiance can be calculated: the total beam, *Q_ℓ,bt_* (unintercepted beam plus down scattered beam), direct beam, *Q_ℓ,b_* (unintercepted beam) and the diffuse flux, *Q_ℓ,d_* [17], [29].
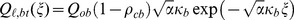
(22)

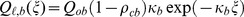
(23)

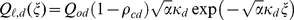
(24)

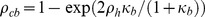
(25)

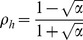
(26)

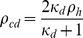
(27)where *α* is absorptivity of leaves for irradiation, *ρ_cb_* and *ρ_cd_* are canopy reflectance for beam and diffuse irradiance respectively with a randomly spherical leaf-angle distribution, *ρ_h_* is canopy reflectance for beam irradiance with a horizontal leaf-angle distribution, and *κ_d_* is an extinction coefficient for diffuse radiation.

The absorbed irradiance in a canopy height (*Q*_ℓ_) consists of the total beam radiation (*Q_ℓ,bt_*) and the diffuse radiation (*Q_ℓ,d_*).
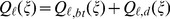
(28)


The total irradiance absorbed by the entire canopy (*Q_c_*) per unit ground area was determined by integrating *Q_ℓ_* over the total *LAI.*

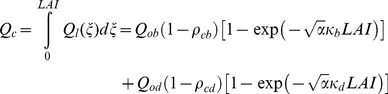
(29)


The irradiance absorbed by the sunlit fraction in a specific canopy height (*Q_ℓ,sl_*) can be given as the sum of direct-beam (*Q_ℓ,b_*), diffuse (*Q_ℓ,d_*) and scattered-beam components (*Q_ℓ,s_*).
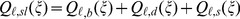
(30)

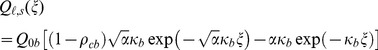
(31)


The irradiance absorbed by the sunlit fraction in the entire canopy (*Q_sl_*) was obtained by integrating *Q_ℓ,sl_* over the total *LAI.*

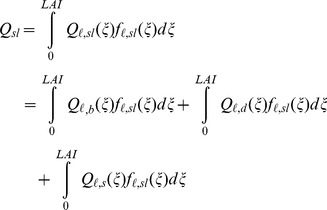
(32)

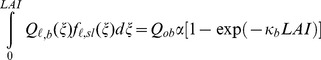
(32a)

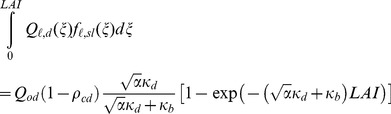
(32b)

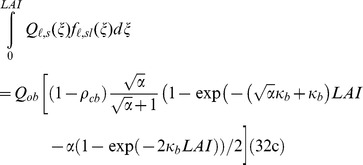
(32c)


The total irradiance absorbed (*Q_c_*) is the sum of the two parts, irradiance absorbed by the separate sunlit (*Q_sl_*) and shade fractions (*Q_sh_*) of the canopy. Thus, *Q_sh_* was calculated as the difference between *Q_c_* and *Q_sl_*.
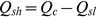
(33)


### 3 Leaf and Canopy Stomatal Conductance

The stomatal conductance is represented by *g_s_* for a single leaf and *G_sc_* for the entire canopy.

#### 3.1. Leaf stomatal conductance

The leaf *g_s_* can be calculated using the Jarvis-Stewart type multiple formulae [30], [31], [32].
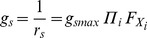
(34)where *g_smax_* is the maximum value of the leaf stomatal conductance and *Fx_i_* is the stress function of the specific environmental variables (*x_i_*), 0≤*Fx_i_*≤1. The original model used short-wave radiation as the light variable. Here we have used the photosynthetically active radiation absorbed by canopy leaves (*Q_a_*) because stomatal aperture is determined by the received visible wavelength radiation, rather than short-wave radiation [17], [18], [33]. In addition, we incorporated the environmental stress impact on *g_s_* by *VPD* and available soil water as follows.



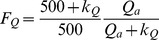
(35)

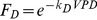
(36)

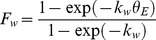
(37)

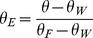
(38)where the *k_Q_*, *k_D_* and *k_w_* are the stress coefficients of *Q_a_*, *VPD* and extractable soil water in the root zone (*θ_E_*), and *θ*, *θ_F_* and *θ_W_* are the measured soil moisture, field capacity and wilting point in the root zone, respectively.

#### 3.2. Big-leaf model of canopy stomatal conductance

The canopy stomatal conductance in the big-leaf model (*G_sc1_*) is estimated by scaling up *g_s_* weighing by the effective *LAI* (*LAI_e_*) as if the canopy is a single big-leaf [16], [18], [32].
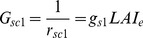
(39)where *g_s1_* is the mean leaf stomatal conductance for the entire big-leaf and can be calculated by Eq. (34) based on the mean absorbed irradiance of the entire canopy using Eq. (29); *LAI_e_* is empirically equal to the actual *LAI* for *LAI*≤2, *LAI*/2 for *LAI*≥4, and 2.0 for others [16].

#### 3.3. Dual-leaf model of canopy stomatal conductance

In the dual-leaf model, *G_sc_* (*G_sc2_*) is calculated by summing the contributions of sunlit and shaded fractions, *G_sl_* and *G_sh_*, respectively, which are scaled up using the associated *g_s_* weighted by their respective fractions of *LAI* [18], [24].
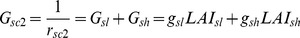
(40)where *g_sl_* and *g_sh_* are the mean leaf stomatal conductance for sunlit and shaded leaves, respectively, and can be calculated by Eq. (34) based on the separate absorbed irradiance using Eqs. (32) and (33). *LAI_sl_* and *LAI_sh_* are *LAI* for sunlit and shaded leaves in the entire canopy, respectively.

Assuming that all leaves in a canopy are randomly distributed, the fraction of sunlit leaves (*f_ℓ,sl_*) in a specific canopy depth declines exponentially with cumulative leaf area (*ξ*) [29], [34].
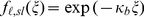
(41)

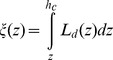
(42)where *κ_b_* is an extinction coefficient for beam radiation, *L_d_* is leaf area density, *z* is height above ground, and *h_c_* is canopy height. *LAI_sl_* is therefore calculated by integrating *f_ℓ,sl_* for the entire canopy.



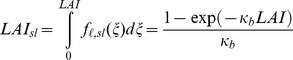
(43)

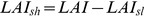
(44)


### 4 Soil Surface Resistance

In this study, *r_ss_* was directly calculated with a function dependent on surface soil water content [35], accounting for the effect of plastic mulching on reduction of soil evaporation by introducing a term for fraction of plastic mulch, *f_m_* [i.e. *r_ss_* is divided by the area of exposed substrate per unit ground area (1−*f_m_*)].
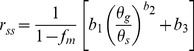
(45)where *θ_g_* is the average soil water content between 0–0.1 m, *θ_s_* is the saturated water content of surface soil, and *b_1_*, *b_2_*, and *b_3_* are the empirical coefficients.

## Materials and Methods

### 1 Experimental Arrangement

The experiments were conducted in an irrigated maize field in 2009 and 2010 at Shiyanghe Experimental Station for Water-saving in Agriculture and Ecology of the China Agricultural University in Gansu Province in northwest China (N 37°52′, E 102°50′, Altitude 1581 m). Grain maize was sown on April 21 and May 2, and harvested on September 28 and September 26 in 2009 and 2010, respectively. The ground was partly mulched with plastic films with a width of 100 cm, and there was bare soil of 65 and 45 cm in width between two film rows in 2009 and 2010, respectively. Maize seed was sown in holes of 5.0 cm diameter under the plastic films, with a row spacing of 50 and 45 cm and a plant spacing of 24 and 23 cm for 2 years. Maize was not sown in the bare soil. This planting scheme had an actual density of 76,300 and 82,500 plants ha^−1^ for 2009 and 2010, respectively. Actual fractions of ground-mulching were ∼0.5 and 0.6, respectively, which were defined as one minus the ratio of the summed surface areas of bare soil and holes to ground area. For the 0–1.0 m soil depth, the soil type was silt loam, with a bulk density of 1.38 g cm^−3^, a field capacity of 0.30 m^3 ^m^−3^, and a wilting point of 0.12 m^3 ^m^−3^. Over the entire growing season, maize was border-irrigated four times, with a total irrigation water amount of 420 mm for both years. The amount of each irrigation event was measured by a water meter. Each irrigation amount was 105 mm on June 15, July 6, July 29 and August 20, 2009, and 105, 120, 90, and 105 mm on June 22, July 27, August 5 and August 29, 2010, respectively.

### 2 Measurements of Evapotranspiration and Soil Evaporation

*λET* was measured using an eddy covariance (EC) system installed in the center of the maize field. The EC consists of a fast response 3D sonic anemometer (CSAT3, Campbell Scientific Inc., UT, USA), a Krypton hygrometer (KH20, Campbell Scientific Inc.) and a temperature and humidity sensor (HMP45C, Vaisala Inc., Helsinki, Finland). All sensors were connected to a data logger (CR5000, Campbell Scientific, Inc.). The sonic anemometer and Krypton hygrometer were installed at a height of 1.0 m over the crop canopy. Net radiation (*R_n_*) was measured by a net radiometer (NR-LITE, Kipp & Zonen, Delft, Netherlands), installed at a height of 3.5 m. Two soil heat fluxes (HFP01, Hukseflux, Delft, Netherlands) were installed at a soil depth of 8.0 cm under the plastic film and bare soil. Soil temperature above each soil heat flux plate was measured using thermocouples at depths of 0.0 cm, 2.0 cm and 6.0 cm. Soil water content from 0–10.0 cm was measured using a soil moisture reflectometer (EnviroSMART, Sentek Sensor Technologies, SA, Australia). Ground heat flux (*G*) was estimated by correcting heat fluxes at 8.0 cm for heat storage above the transducers. The heat storage was determined from changes in soil temperature and moisture above the transducers. Based on the covariance of the 10 Hz air temperature and specific humidity with vertical wind velocity, the latent heat flux in 30 min durations was computed using the eddy covariance methodology with the CarboEurope recommendations [36]. Daytime *λET* was adjusted by the Bowen-ratio forced closure method, and nighttime *λET* was adjusted using the filtering interpolation method as proposed by Ding et al. [37].

Soil evaporation (*E_s_*) was measured by the micro-lysimeter. Eight micro-lysimeter cylinders, made from PVC tubes with a diameter of 10 cm and height of 20 cm, were installed in bare soil between two plastic film rows. The cylinders were weighed at 20∶00 every day by an electric scale with a precision of 0.1 g. The micro-lysimeters were reinstalled within one day after each irrigation and heavy rain. *E_s_* at the field scale can be calculated by weighting the fraction of ground-mulching (*f_m_*) from the following equation.
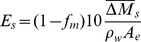
(46)where 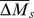 is the mean weight change of micro-lysimeter every day, *A_e_* is the cross sectional area of the micro-lysimeters (78.5 cm^2^ here), *ρ_w_* is water density (1.0 g cm^−3^) and 10 is a conversion factor for changing units from cm to mm.

### 3 Other Measurements

Solar radiation, precipitation, air temperature, relative humidity and wind speed were measured with a standard automatic weather station (Hobo, Onset Computer Corp., USA) at a height of 2.0 m above the ground. Volumetric soil water content in the root zone (*θ_rz_*) was measured with PVC access tubes using the portable device Diviner 2000 (Sentek Sensor Technologies). Measurements were made at intervals of 0.1 m with a maximal soil depth of 1.0 m at intervals of 3–5 days. Additional samplings were conducted before and after irrigation events, as well as after rainfall events. The measurements were calibrated by oven drying of soil samples. Interpolation was applied between consecutive irrigations to determine *θ_r_* for each day. In addition, two sets of ECH2O probes (Decagon Devices Inc., Pullman, WA, USA) were added to monitor soil moisture at 30 min intervals in 2010.

Ten maize plants were randomly selected to measure leaf length and width, and height at intervals of ∼10 days during the growing season. Leaf area was calculated by summing the rectangular area of each leaf (leaf length×maximum width) multiplied by a factor of 0.74, a conversion factor obtained by analyzing the ratio of the rectangular area to the real area measured by an AM300 (ADC BioScientific Ltd., UK). *LAI* is defined as maize green leaf area per unit ground area. The daily *LAI* was obtained by linear interpolation.

Leaf-scale physiological measurements were performed to derive the stomatal conductance model parameters. A LI-6400 portable photosynthesis system (Li-Cor Inc., Lincoln, NE, USA) was used to measure leaf stomatal conductance on the first fully expanded leaf, which is the fourth leaf counted from the top of the shoot. The diurnal measurements of leaf gas exchange were performed once every 2 h from 8∶00 to 18∶00 on six sunny days in 10 randomly selected maize plants. Care was taken to keep leaves in their natural positions during measurement. The response of leaf stomatal conductance to varying PAR was measured at 30°C and at a CO_2_ concentration of 400 mol mol^−1^ on 29 August, 2009. Measurements were taken at PAR levels of 2000, 1600, 1300, 1000, 800, 600, 400, 200, 100, 50, 20, and 0 µmol m^−2 ^s^−1^. The stomatal light-response curve was fit by a rectangular hyperbola to obtain the parameter values of *k_Q_* using the Jarvis-Stewart model.

### 4 Model Performance

Half-hourly *G_sc_* and *λET* were calculated using the big-leaf and dual-leaf models Eqs. (39) and (40) with the dual-source equation based on the half-hourly measured meteorological data. The *LAI* and soil water were set as constants at the half-hourly time scale. Daily *λET* was calculated using Eqs. (39) and (40) with the dual-source equation based on the measured average daily meteorological data. We evaluated the two models by comparing with measurements taken over an irrigated maize field.

The parameters in the Jarvis-Stewart model were obtained using measurements of the stomatal light-response curve and the diurnal leaf gas exchange calculated by non-linear least-squares analysis (SPSS 13.0, SPSS Inc., Chicago, IL, USA). There were ∼15 days with no crop cover before the emergence of maize, providing the opportunity to parameterize the empirical coefficients in the soil surface resistance model using the flux observations.

The coefficient of determination (R^2^), root mean square error (RMSE) and the Willmott’s index of agreement (d) were used to evaluate model performance [38].

## Results

### 1 Model Parameter Estimation and Sensitivity

From the stomatal light-response curve, we obtained best-fitting estimates of *k_Q_* by non-linear least-squares analysis using the Jarvis-Stewart model (Table 1). The stress coefficients of *VPD* and *θ_E_*, *k_D_* and *k_w_*, were optimized using the diurnal measurements of leaf gas exchange (Table 1). Soil surface resistance (*r_ss_*) was calculated by inverting the flux-resistance equation for the case of no crops [12]. Based on the relationship between *r_ss_* and relative soil water content (*θ_g_*/*θ_s_*) of the top soil, the best-fitting parameters of *b_1_*, *b_2_* and *b_3_* were obtained (Table 1).

**Table 1:** The parameters used in the dual-source dual-leaf model.

Symbol	Description	Value	Units	Sources
*b_1_*	Parameter in soil resistance model	15.2	s m^−1^	Fitted in this study
*b_2_*	Parameter in soil resistance model	−5.8	-	Fitted in this study
*b_3_*	Parameter in soil resistance model	88.7	s m^−1^	Fitted in this study
*c_d_*	Mean drag coefficient for the individual vegetative elements	0.1	-	Meyers and Paw [42]
*d_l_*	Characteristic leaf dimension	0.068	m	Measured in this study
*G_L_*	Spherical leaf angle distribution	0.5	-	Campbell and Norman [17]
*g_smax_*	Maximum value of the leaf stomatal conductance	7.5	mm s^−1^	Fitted in this study
*k*	von Karman’s constant	0.41	-	Brutsaert [41]
*k_Q_*	Stress coefficients of thephotosynthetically active radiationin the stomatalconductance model	150	W m^−2^	Fitted in this study
*k_D_*	Stress coefficients of the vapor pressure deficit in the stomatal conductancemodel	0.2	kPa^−1^	Fitted in this study
*k_w_*	Stress coefficients of the extractable soil water in the root zone in the stomatal conductance model	7.5	-	Fitted in this study
*z_0s_*	Effective roughness length of the soil substrate	0.01	m	Shuttleworth and Wallace [14]
*α*	Absorptivity of leaves of irradiation	0.8	-	Monteith and Unsworth [12]
*κ_d_*	Extinction coefficient for diffuse radiation	0.7	-	Campbell and Norman [17]
*τ_a_*	Atmospheric transmittance	0.72	-	Brutsaert [41]
*f_a_*	Forward scattering coefficient of PAR in the atmosphere	0.43	-	Brutsaert [41]
*f_m_*	Fraction of ground mulched by plastic film	0.5/0.6^a^	-	Measured in this study

a 0.5/0.6 is the fraction of ground mulched by plastic film in 2009 and 2010, respectively.

**Figure 1:**
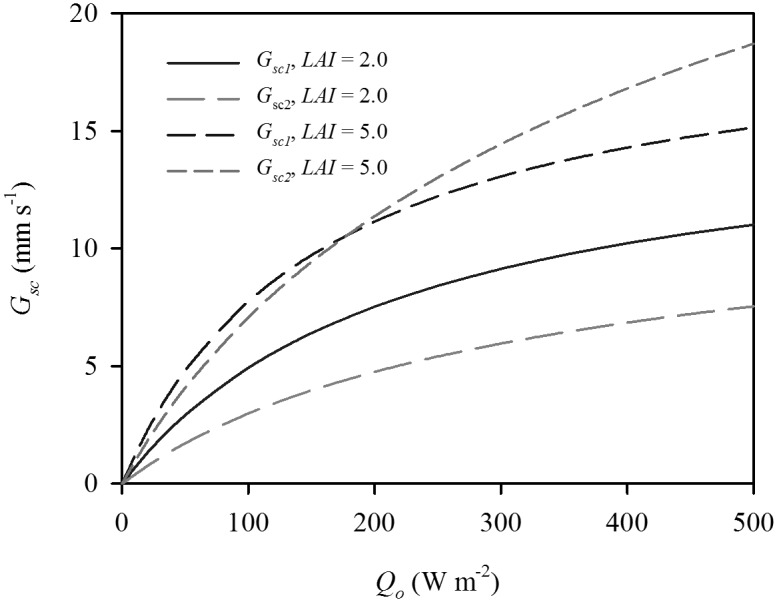
Response of canopy stomatal conductance (*G_sc_*) to incident PAR above the canopy (*Q_o_*) at different leaf area indices (*LAI*). *G_sc1_* and *G_sc2_* are calculated by the big-leaf and dual-leaf canopy stomatal resistance models, respectively.

**Figure 2:**
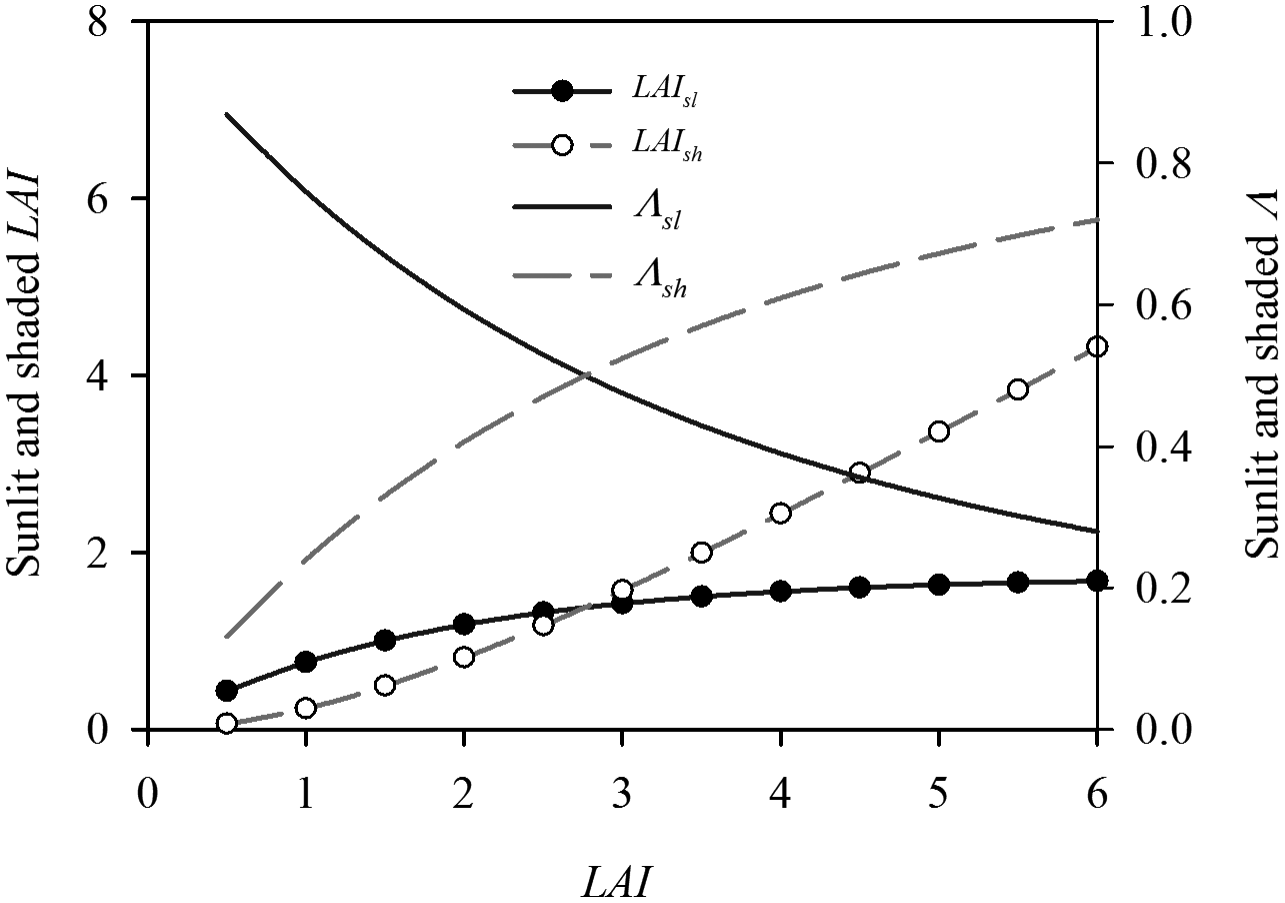
Variation in the sunlit and shaded leaf area indices (*LAI_sl_* and *LAI_sh_*) versus different *LAI*. The ratios of *LAI_sl_* and *LAI_sh_* to *LAI* (*Λ_sl_* and *Λ_sh_*) are also shown.

The big-leaf model estimates did not closely match the response of *G_sc_* to incident PAR above the canopy (*Q_o_*) predicted by the dual-leaf model (Fig. 1). At lower *LAI* (2.0), the big-leaf model overestimated *G_sc_* by up to 47.4% at an intermediate irradiance (300 W m^−2^). At higher *LAI* (5.0), the big-leaf model overestimated *G_sc_* when *Q_o_*<200 W m^−2^ and underestimated *G_sc_* when *Q_o_*>200 W m^−2^. The sensitivity of the dual-leaf model was further analyzed by investigating the variations of the sunlit and shaded leaf area index (*LAI_sl_* and *LAI_sh_*) against the different *LAI* (Fig. 2). *LAI_sl_* approached a maximum of 1.6 when *LAI*≥3.0, while *LAI_sh_* almost linearly increased as *LAI* increased. As a result, the ratios of *LAI_sl_* and *LAI_sh_* to *LAI* (*Λ_sl_* and *Λ_sh_*) nonlinearly decreased and increased, respectively, as *LAI* increased.

**Figure 3:**
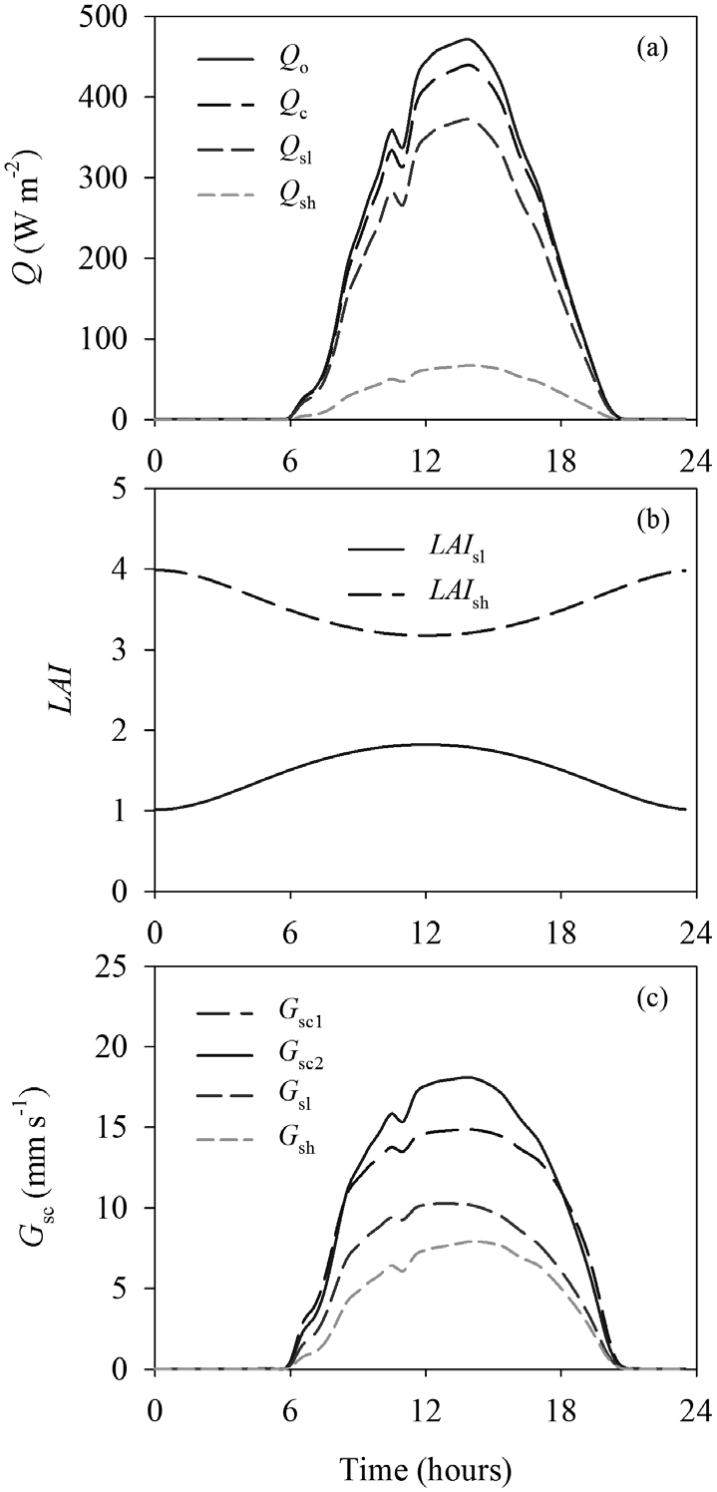
Diurnal variations of (a) modeled irradiance, (b) leaf area index and (c) canopy stomatal conductance at leaf area index (*LAI*) = 5.0. *Q_o_* is the total irradiance above the canopy, *Q_c_* is the irradiance absorbed by the entire canopy, and is separated into the irradiance absorbed by the sunlit leaves of the canopy (*Q_sl_*) and irradiance absorbed by the shaded leaves of the canopy (*Q_sh_*). *LAI_sl_* and *LAI_sh_* are the sunlit and shaded fractions of *LAI*, respectively. *G_sc1_* and *G_sc2_* are the canopy stomatal conductance calculated by the big-leaf and dual-leaf models, respectively; *G_sc2_* is separated into the sunlit and shaded canopy stomatal resistance, *G_sl_* and *G_sh_*, respectively.

The diurnal variations of modeled irradiance absorbed by the entire canopy (*Q_c_*) and its separation into sunlit and shaded fractions (*Q_sl_* and *Q_sh_*) are shown in Fig. 3a at higher *LAI* = 5.0, using the measured diurnal courses of meteorological and environmental variables from June 23, 2009. *Q_c_*, *Q_sl_* and *Q_sh_* exhibited the typical diurnal patterns, while *Q_sh_* had a lower magnitude than *Q_sl_* throughout the day. The average *Q_sl_* accounted for 84.2% of the *Q_c_*. The partitioning of leaves into sunlit and shaded fractions continually changes throughout the day (Fig. 3b). *LAI_sl_* was a convex parabola, while *LAI_sh_* was a concave parabola. The magnitude of *LAI_sh_* was greater than *LAI_sl_* even during midday when the solar zenith angle is lowest at the higher *LAI*, which is consistent with the result in Fig. 2. The diurnal distributions of *G_sc_* calculated by the big-leaf model (*G_sc1_*) and dual-leaf model (*G_sc2_*) and its separation between sunlit and shaded fractions (*G_sl_* and *G_sh_*) are presented in Fig. 3c. *G_sc1_* was significantly lower than *G_sc2_* through most of the day at the higher *LAI*. *G_sh_* showed a pattern similar to *G_sl_*, while the magnitude of *G_sh_* was lower than *G_sl_*. The maximum difference between them occurred at the midday around 11∶00.

### 2 Comparisons of Canopy Stomatal Conductance by Big-leaf and Dual-leaf Models

**Figure 4:**
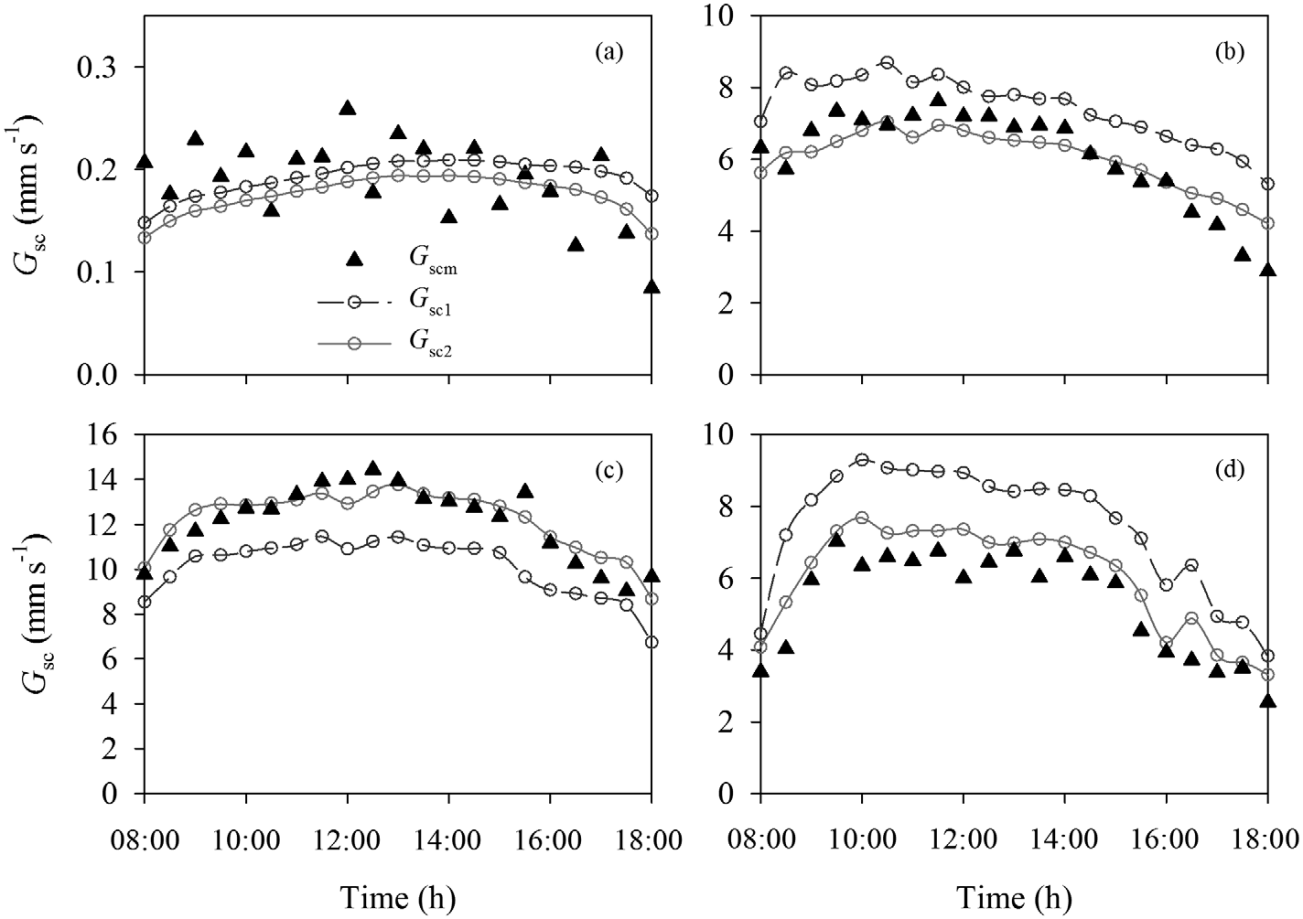
Diurnal variations of half-hourly canopy stomatal conductance (*G_sc_*) calculated by the big-leaf (*G_sc1_*) and dual-leaf models (*G_sc2_*), and measured *G_sc_* (*G_scm_*) inverted by the S-W model, respectively for four typical clear-sky days in different growth stages of maize in 2009. Leaf area index (*LAI*) was 0.16, 2.62, 5.38 and 2.99, on (a) May 22, (b) June 23, (c) July 27, and (d) September 17, respectively.

**Figure 5:**
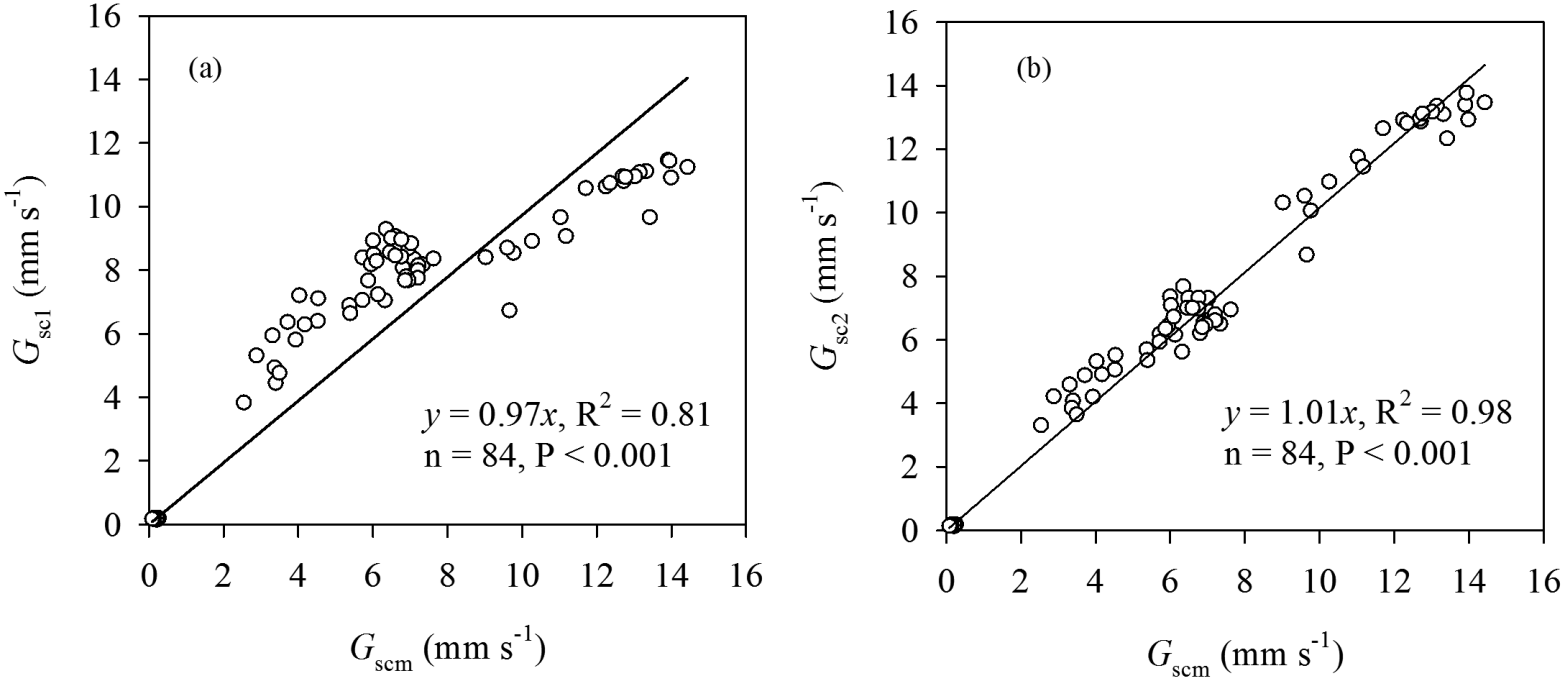
Relationship between canopy stomatal conductance (*G_sc_*) estimated by the big-leaf (*G_sc1_*) and dual-leaf models (*G_sc2_*), and measured *G_sc_* (*G_scm_*) inverted by the S-W model, respectively for four typical clear-sky days in different growth stages of maize in 2009.

Diurnal patterns of *G_sc_* calculated by the big-leaf and dual-leaf models (*G_sc1_* and *G_sc2_*, respectively) and the measured *G_sc_* (*G_scm_*) calculated by using Eq. (17) are shown in Fig. 4 for four typical clear-sky days in different growth stages of maize in 2009. The diurnal courses of the *G_sc1_ and G_sc2_* were similar to the *G_scm_*, but there were differences in magnitude. *G_sc1_* was significantly lower than *G_scm_*, while *G_sc2_* closely matched *G_scm_* at higher *LAI* (Fig. 4c). *G_sc1_* was higher than *G_scm_* at the lower *LAI* where there was still good agreement between *G_sc2_* and *G_scm_* values (Fig. 4b and d). Although the slope of the linear regression between *G_sc1_* and *G_scm_* was 0.97, *G_sc1_* was overestimated at the lower *G_scm_* and underestimated at the higher *G_scm_* (Fig. 5a), as shown by the lower R^2^ of 0.81, greater RMSE of 1.7047 mm s^−1^ and lower d of 0.9563, indicating that the big-leaf model yielded large errors for estimating *G_sc_*. In contrast, the slope of the linear regression between *G_sc2_* and *G_scm_* was 1.01, with an R^2^ of 0.98, RMSE of 0.6120 mm s^−1^ and d of 0.9951, indicating that there was good data-model agreement between predictions and measurements (Fig. 5b).

### 3 Comparisons of Crop Evapotranspiration by Big-leaf and Dual-leaf Models

**Figure 6:**
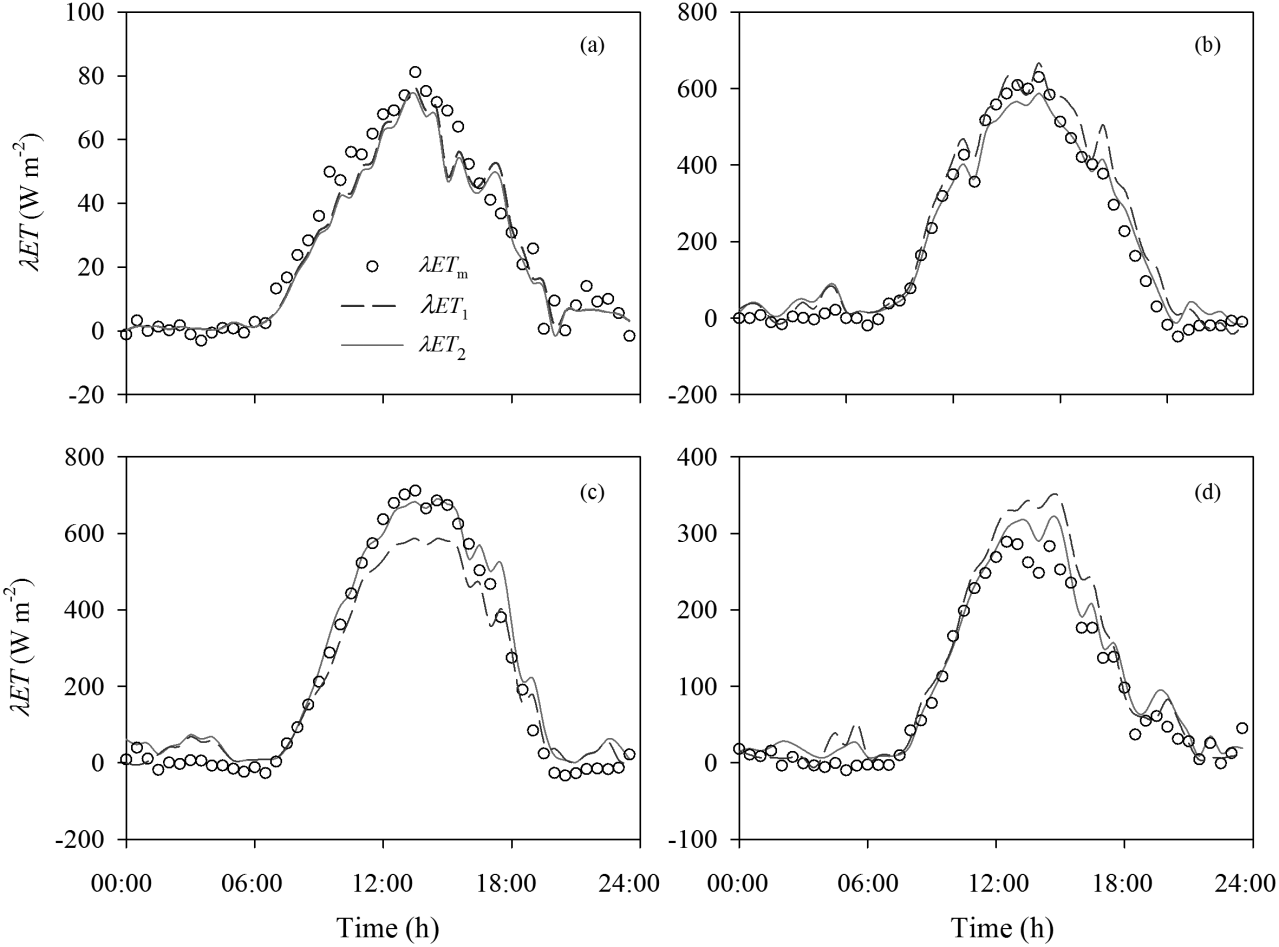
Diurnal variations of half-hourly crop evapotranspiration (*λET*) calculated by the dual-source with the big-leaf (*λET_1_*) and dual-leaf models (*λET_2_*), and measured *λET* (*λET_m_*), respectively for four typical clear-sky days in different growth stages of maize in 2009. Leaf area index (*LAI*) was 0.16, 2.62, 5.38 and 2.99, on (a) May 22, (b) June 23, (c) July 27, and (d) September 17, respectively.

Diurnal variations of half-hourly *λET* calculated by the dual-source big-leaf (*λET_1_*) and dual-leaf models (*λET_2_*), and measured *λET* (*λET_m_*), respectively are presented for four typical clear-sky days in different growth stages of maize in 2009 (Fig. 6). The diurnal patterns of estimated *λET* were similar to the measurements. *λET_1_* was overestimated at lower *LAI*, and underestimated at higher *LAI*. The linear regression presented that *λET_1_* was overestimated by 8.7% (R^2^ = 0.97) and 19.7% (R^2^ = 0.96) for *LAI* = 2.62 and 2.99, respectively (Fig. 6b and d). *λET_1_* was underestimated by 13.9% (R^2^ = 0.97) for *LAI* = 5.38 (Fig. 6c). In contrast, *λET_2_* had a good agreement with measurements for differing *LAI*, with a linear slope of 1.01 and an R^2^ of 0.97.

The irrigation scheduling and mulching fractions in 2009 and 2010 were different (See section 3.1), which yielded different *LAI* and soil water regimes for the two years (See Figure 1 and Table 1 in Ding et al.[39]). The maximum and averaged values of *LAI* respectively were 5.4 and 3.1 for 2009, and 4.7 and 2.7 for 2010. The extractable soil water in the root zone (*θ_E_*) was significantly different between the two years. Before the first and second irrigation events in 2010, there were 9 and 12 days of *θ_E_* below 50% total available water in the root zone (*TAW*), which was regarded as a threshold of crop water stress [13]. Conversely, most *θ_E_* was higher than 50% of *TAW* in 2009. All of these differences led to differing *λET* and its components, which provided a good dataset to test the big-leaf and dual-leaf models over two different hydrometeorological and management strategies.

**Figure 7:**
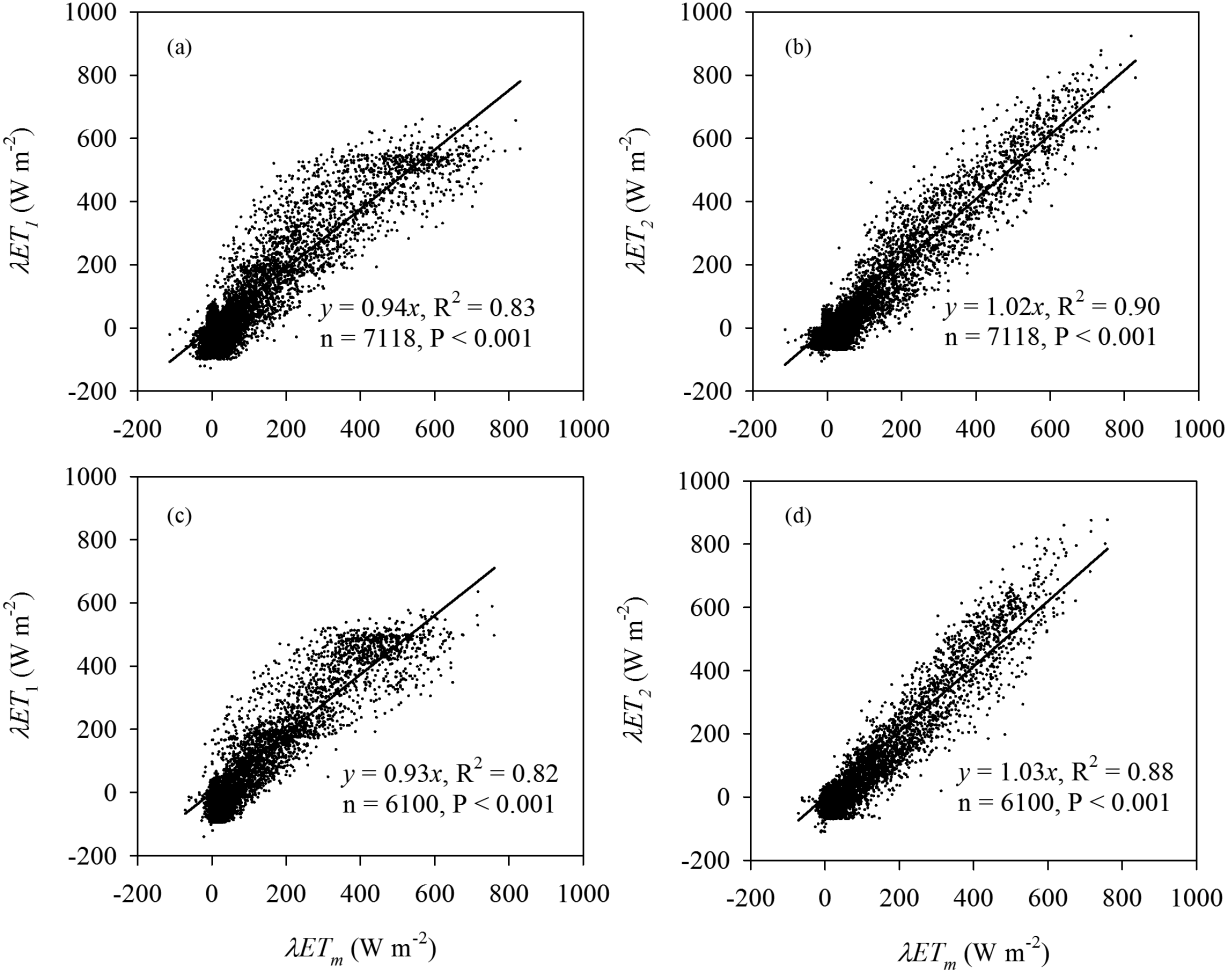
Comparison between half-hourly estimated evapotranspiration by the dual-source with the big-leaf (*λET_1_*) and dual-leaf models (*λET_2_*) versus measured *λET* by eddy covariance (*λET_m_*) during the entire growth period of maize in (a and b) 2009 and (c and d) 2010.

The scatterplots of half-hourly *λET* exhibited that *λET_1_* was overestimated for lower values and underestimated for higher values, respectively (Fig. 7a and 7c). Total *λET_1_* was underestimated, with a slope of linear regression of 0.94 (R^2^ = 0.83) and 0.93 (R^2^ = 0.82), respectively, for 2009 and 2010. RMSE was 72.22 and 70.97 W m^−2^, and d was 0.9521 and 0.9472 for 2009 and 2010, respectively (Table 2). In contrast, there was good data-model agreement between measurements and estimated half-hourly *λET_2_* in 2009 and 2010 (Fig. 7b and 7d). The slopes of linear regressions between the estimates and measurements were 1.02 and 1.03, with R^2^ of 0.90 and 0.88, RMSE of 58.06 and 62.31 W m^−2^ and d of 0.9706 and 0.9626 for 2009 and 2010, respectively (Table 2). Daily estimated *λET_2_* enhanced data-model agreement, with R^2^ of 0.91 for the 2 years despite the linear slopes were the same as those of half-hourly values (data not shown). The statistical test showed that the slopes were not significantly different with one (P = 0.114 and 0.092), and the intercepts were not significantly different with zero (P = 0.215 and 0.174) for the 2 years.

**Table 2:** Comparison of measured and estimated evapotranspiration (*λET*) and soil evaporation (*E_s_*) during the growth periods of maize in 2009 and 2010.

Years	Time-scales	Models	Average values		Linear regression equation with zero intercept	R^2^	RMSE (mm s^−1^)	d
			Measurements (*x*)	Estimates (*y*)				
2009	Half-hourly *λET*	Big-leaf	102.4	79.3	*y* = 0.94*x*	0.83	72.22	0.9521
		Dual-leaf	102.4	87.6	*y* = 1.02*x*	0.90	58.06	0.9706
	Daily *E_s_*	Big-leaf	0.44	0.43	*y* = 0.95*x*	0.63	0.1198	0.9036
		Dual-leaf	0.44	0.46	*y* = 1.02*x*	0.68	0.1220	0.9129
2010	Half-hourly *λET*	Big-leaf	107.0	75.2	*y* = 0.93*x*	0.82	70.97	0.9472
		Dual-leaf	107.0	82.9	*y* = 1.03*x*	0.88	62.31	0.9626
	Daily *E_s_*	Big-leaf	0.45	0.44	*y* = 0.93*x*	0.64	0.1593	0.9054
		Dual-leaf	0.45	0.46	*y* = 1.01*x*	0.73	0.1565	0.9218

Note: R^2^ is the coefficient of determination, RMSE is the root mean square error, and d is the Willmott’s index of agreement. The units of half-hourly and daily values are W m^−2^ and mm d^−1^, respectively.

**Figure 8:**
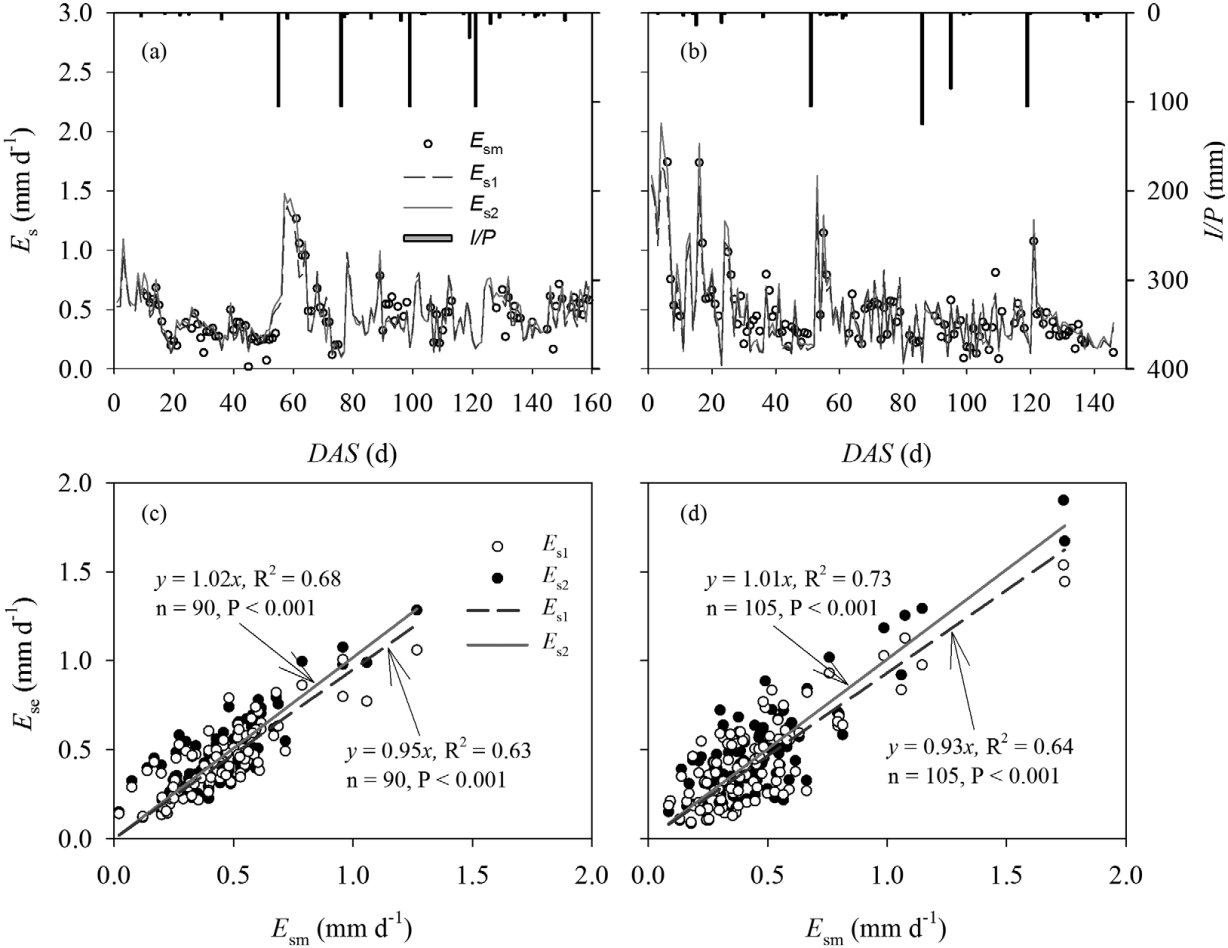
Comparison between daily estimated soil evaporation (*E_se_*) by the dual-source with the big-leaf (*E_s1_*) and dual-leaf models (*E_s2_*) versus measurements (*E_sm_*) during the entire growth period of maize in 2009 and 2010. Seasonal variations of the estimated and measured *E_s_* against days after sowing (*DAS*) are presented in (a) and (b). The linear regressions between them are presented in (c) and (d).

Seasonal variations of daily estimated and measured *E_s_* using Eq. (15) combined with Eqs. (39) and (40) are presented in Fig. 8 for 2009 and 2010. Both the dual-source big-leaf and dual-leaf models could capture the variability of *E_s_* even when irrigation or precipitation occurred and when the canopy partially covered the ground during the initial growth stages. However, daily values of *E_s1_* were underestimated at the early and late stages and overestimated at the middle stage (Fig. 8a and b). In general, total *E_s1_* was underestimated by 5% and 7% for 2009 and 2010, respectively (Fig. 8c and d). In contrast, there was satisfactory data-model agreement between predicted and measured *E_s_* using the DSDL model for the two years. The slopes of linear regressions between estimates and measurements were 1.02 (R^2^ = 0.68) and 1.01 (R^2^ = 0.73), with RMSE of 0.1220 and 0.1565 mm d^−1^ and d of 0.9129 and 0.9218 for 2009 and 2010, respectively (Table 2).

## Discussion

In this paper, we have extended the big-leaf model by developing a dual-leaf model. The dual-leaf model presented here is an improvement over the previous big-leaf model, as more realistic non-uniform vertical profiles of radiation and stomatal conductance are now incorporated into the model. The penetration of beam radiation, its variation and the dynamics of sunlit and shaded *LAI* throughout the day all affect the ability of the big-leaf model to simulate diurnal changes in *G_sc_* [18], [29]. In the dual-leaf model, these canopy features can be explicitly incorporated by dividing the canopy into sunlit and shaded fractions and modeling each fraction of *G_sc_* by scaling up the respective stomatal conductance separately. It is more complex than the big-leaf model, but the dynamic partitioning of *LAI* and irradiance between sunlit and shaded leaves has further reduced the errors associated with simplifying the leaves to only a big-leaf using either the total or empirically effective *LAI*.

The ability of the dual-leaf model was examined by comparing the estimated values and actual measurements. In contrast to significant errors by the big-leaf model, the dual-leaf model accurately reproduced the variation of *G_sc_* (Fig. 4 and 5). One reason the dual-leaf model works so well is that it accommodates the nonlinear response of stomata to light [25], [40]. Stomata-light responses of leaves can vary with depth in the canopy and this variation can be incorporated by partitioning the canopy into several layers and estimating the sunlit and shaded leaf fractions in each layer [20], [34]. However, usually this is not necessary and a single, representative light response curve can be used for the entire canopy [17], [40]. In the dual-leaf model, the entire *G_sc_* may be calculated by summing contributions of sunlit and shaded leaves. These two contributions are added separately because sunlit leaves will be light-saturated while shaded leaves will be in the linear portion of the light-stomata relationship [18], [33]; thus *G_sc_* is not proportional to average light levels [18]. Because of the nonlinear relationship between stomatal conductance and PAR, the predicted *G_sc_* will be overestimated when the average absorbed PAR is used to scale up the leaf stomatal conductance for the entire canopy as in the big-leaf model [17], [40]. On the other hand, the *G_sc_* is underestimated when the effective *LAI* is used to scale up the leaf stomatal conductance at higher *LAI* ((Fig. 4c). Since the nonlinear relationship was considered and the sunlit-shaded method was introduced in the dual-leaf model, estimates using the dual-leaf model closely match the measured *G_sc_* (Fig. 4 and 5).

The performance of the DSDL for estimating *λET* was investigated. The DSDL is robust for estimating *λET* over a range of canopy leaf areas and environmental variables (Fig. 6, Fig. 7 and Table 2). The good data-model agreement indicated the strengths of the DSDL model as a model framework and the reference for validating other approaches of calculating the *G_sc_* using the dual-leaf model. Our framework of modeling *λET* also provided a soil evaporation estimation model Eq.(15) with a modified soil surface resistance term, which is useful for enhancing crop production by reducing the *E_s_* fraction of *λET* [10], [15].

The DSDL model is physically process-based, yet sufficiently simple to be effectively parameterized. The dual-leaf model requires only four additional equations, Eqs (32), (33), (43) and (44), beyond those required in the model of leaf stomatal conductance, to calculate the *LAI* and absorbed irradiance of the sunlit and shaded leaves. This simplicity makes it attractive for incorporation into in crop models, land surface schemes, and regional or global water cycle studies [29], [40]. This model can also be used to assess effects of climate change on crop ecophysiology.

## Conclusions

In this paper, a dual-leaf model for scaling-up stomatal conductance from the leaf to the canopy level was developed through the dynamic partitioning of the leaf area index and irradiance between sunlit and shaded leaves. In the model, canopy stomatal conductance was calculated by dividing the canopy into sunlit and shaded fractions and each fraction was modeled separately based on the absorbed irradiances. The dual-leaf model provided estimates of *G_sc_* which were nearly the same as measurements, and were significantly more accurate than those of the big-leaf model. Our results showed excellent agreements between *λET* measurements gathered by the eddy covariance technique over an irrigated maize field during 2009 and 2010 under two different hydrometeorological and management conditions, and estimates of *λET* using the DSDL. The framework of the model can also satisfactorily estimate soil evaporation. Our proposed model provides an alternative approach to calculate *λET*, which is simple and attractive for incorporation into other comprehensive crop models.

## Supporting Information

Appendix S1 Resistances calculations and solar geometry.
(DOCX)
