# Supplementary material for: Scaling Up Stomatal Conductance from Leaf to Canopy Using a Dual-Leaf Model for Estimating Crop Evapotranspiration
Source: PLoS One. 2014 Apr 21;9(4):e95584. doi: 10.1371/journal.pone.0095584 (PMC3994067; doi:10.1371/journal.pone.0095584)
Supplement: Appendix S1 — Resistances calculations and solar geometry. (DOCX) [file pone.0095584.s001.docx]

# Appendix S1. Resistances calculations and solar geometry

# Resistances calculations in the dual-source model

The aerodynamic resistances *r_as_* and *r_aa_* are calculated by integrating the eddy diffusion coefficients from the soil surface to the level of the preferred sink of momentum in the canopy, and from there to the reference height [26]. This empirically and simply simulates a second-order closure theory of in-canopy turbulence.

$$r_{aa}=\frac{1}{ku_{*}}\ln\left(\frac{z_{m}-d_{0}}{h_{c}-d_{0}}\right)+\frac{h_{c}}{\kappa_{m}K_{h}}\left\{\exp\left[\kappa_{m}\left(1-\frac{d_{0}+z_{0}}{h_{c}}\right)\right]-1\right\}$$

$$r_{as}=\frac{h_{c}\exp(\kappa_{m})}{\kappa_{m}K_{h}}\left\{\exp\left(\frac{-\kappa_{m}z_{0s}}{h_{c}}\right)-\exp\left[-\kappa_{m}\left(\frac{d_{0}+z_{0}}{h_{c}}\right)\right]\right\}$$

where *k* is von Karman’s constant, *z_m_* is the reference height of measurement, *u_*_* is the friction velocity, *K_h_* is the eddy diffusion coefficient at the top of the canopy, *κ_m_* is the extinction coefficient of the eddy diffusion *d_0_* is the zero plane displacement, *z_0_* is the roughness lengths governing the transfer of momentum, and *z_0s_* is the effective roughness length of the soil substrate. Brutsaert [41] indicated that *κ_m_* = 2.5 when *h_c_* < 1 m, and *κ_m_* = 4.25 when *h_c_* > 10 m. In our study, *κ_m_* was obtained through linear interpolation.

$$K_{h}=ku_{*}(h_{c}-d_{0})$$

$$u_{*}=ku/\ln\left(\left(z_{m}-d_{0}\right)/z_{0}\right)$$

$$d_{0}=1.1h_{c}\ln\left(1+X^{1/4}\right)$$

$$z_{0}=\left\{\begin{matrix} z_{0s}+0.3h_{c}X^{1/2}\text{ 0<}X\text{<}0.2 \\ 0.3h_{c}\left(1-d_{0}/h_{c}\right)\text{ 0.2<}X\text{<}1.5 \end{matrix}\right.$$

$$X=c_{d}LAI$$

where *c_d_* is the effective value of the mean drag coefficient for the individual vegetative elements making up the canopy [26,42].

The canopy boundary layer resistance *r_ac_* was calculated as Shuttleworth and Gurney [26]:

$$r_{ac}=\frac{r_{b}}{2LAI}$$

$$r_{b}=\frac{100}{\kappa_{m}}(\frac{d_{l}}{u_{h}})/[1-\exp(-\frac{\kappa_{m}}{2})]$$

where *r_b_* is the mean boundary layer resistance, determined from the wind speed at the top of canopy (*u_h_*), the characteristic leaf dimension, and the extinction coefficient of the eddy diffusion (*κ_m_*).

# Solar geometry

Solar zenith angle (*ζ*) was calculated as in Allen et al. [13]:

where *φ* is latitude, *δ* is solar declination, and *ω* is hour angle. The units for the three variables are radian.

$$\delta=0.409\sin\left(\frac{2\pi}{365}J-1.39\right)$$

$$\omega=\frac{\pi}{12}\left[\left(t+0.06667\left(L_{z}-L_{m}\right)+S_{c}\right)-12\right]$$

$$S_{c}=0.1645\sin(2b)-0.1255\cos(b)-0.025\sin(b)$$

$$b=\frac{2\pi(J-81)}{364}$$

where *J* is the number of the day in the year, *t* is the standard clock time at the midpoint of the period, *Lz* is the longitude of the center of the local time zone, *L_m_* is the longitude of the measurement site, and *S_c_* is the seasonal correction for solar time.
